# Role of Lysosomal Acidification Dysfunction in Mesenchymal Stem Cell Senescence

**DOI:** 10.3389/fcell.2022.817877

**Published:** 2022-02-07

**Authors:** Weijun Zhang, Jinwu Bai, Kai Hang, Jianxiang Xu, Chengwei Zhou, Lijun Li, Zhongxiang Wang, Yibo Wang, Kanbin Wang, Deting Xue

**Affiliations:** ^1^ Department of Orthopaedics, Second Affiliated Hospital, Zhejiang University School of Medicine, Zhejiang University, Hangzhou, China; ^2^ Institute of Orthopaedics, School of Medicine, Zhejiang University, Hangzhou, China

**Keywords:** mesenchymal stem cells, senescence, lysosomal acidification, V-ATPase, pH

## Abstract

Mesenchymal stem cell (MSC) transplantation has been widely used as a potential treatment for a variety of diseases. However, the contradiction between the low survival rate of transplanted cells and the beneficial therapeutic effects has affected its clinical use. Lysosomes as organelles at the center of cellular recycling and metabolic signaling, play essential roles in MSC homeostasis. In the first part of this review, we summarize the role of lysosomal acidification dysfunction in MSC senescence. In the second part, we summarize some of the potential strategies targeting lysosomal proteins to enhance the therapeutic effect of MSCs.

## Introduction

Mesenchymal stem cells (MSCs) are pluripotent stem cells with self-renewal (Pittenger et al., 1999), immunosuppressive (Bartholomew et al., 2002), and anti-inflammatory capabilities (Uccelli et al., 2008). MSCs were first extracted from mouse bone marrow by Friedenstein in 1976 when he referred to them as clonogenic fibroblast precursor cells (CFU-F) (Friedenstein et al., 1976). It was not until 1991 that Caplan first defined these cells as mesenchymal stem cells (MSCs) (Caplan, 1991). In 1995, Lazarus et al. completed the world’s first clinical trial of MSCs therapy (Lazarus et al., 1995). As of November 2020, 1,025 clinical trials based on MSC therapies have been registered at FDA. gov (Zhang et al., 2021) (clinicaltrials.gov). However, the therapeutic effects of MSCs given to humans are not as robust as preclinical studies have shown, and most clinical-stage MSC therapies fail to meet the primary efficacy endpoint. To date, only 10 MSC-based products have been approved by regulatory authorities worldwide (Levy et al., 2020).

*In vitro* aging of stem cells severely affects its therapeutic efficacy. These “*in vitro* aged” cells exhibit abnormal morphology, skewed differentiation potential, diminished expression of surface markers, downward migration and antioxidant capacity (Wagner et al., 2008; Geissler et al., 2012; Lunyak et al., 2017; Yang et al., 2018). Diminished autophagic activity and lysosomal function play an important role in these age-related manifestations (Cuervo et al., 2005). With recent advances in the understanding of lysosomal function, new opportunities for treatment by specifically targeting lysosomes are beginning to emerge (Bonam et al., 2019). Lysosomes degrade intracellular pathogens, as well as damaged organelles and proteins, through the autophagic pathway. Lysosomes must be able to respond rapidly with enhanced or diminished function to a variety of metabolic conditions (Ballabio and Bonifacino, 2020). Therefore, depending on the disease context, activation, or inhibition of different components of the lysosome may represent potential pharmacological strategies.

Regulation of lysosomal acidification is an emerging direction in MSCs-based therapy (Ruckenstuhl et al., 2014). Therapeutic strategies targeting lysosomes in autoimmune disorders and neurodegenerative diseases have been described in great detail by Srinivasa Reddy Bonam et al. (Bonam et al., 2019). In this paper, we focus on the relationship between lysosomal acidification and senescence. We assemble information from relevant studies to demonstrate the association between lysosomal acidity and the aging process, as well as highlight the most recent research on lysosomal acidification control, in the hopes of providing some insight into the entire MSC aging process.

## Mechanism of Lysosomal Acidification

Lysosome was first discovered in the 1950s by Christian de Duve et al. (De Duve et al., 1955). It is a membrane-bound vesicle containing more than 60 hydrolytic enzymes that break down proteins, lipids, nucleic acids, and polysaccharides. In addition to degradation, lysosomes are involved in many other cellular processes, including nutrient sensing (Shin and Zoncu, 2020), metabolic signaling (Sancak et al., 2010), chromatin processing (Ivanov et al., 2013), and plasma membrane repair (Morgan et al., 2011). Each of these behaviors is influenced by the internal pH of the lysosome, which is maintained in the 4.5–5 pH range by the vacuolar H^+^-ATPases (V-ATPases) and the counterion transporter, which can be either a cation (moving out of the lysosome) or an anion (moving into the lysosome) (Steinberg et al., 2010; Mindell, 2012).

**FIGURE 1:**
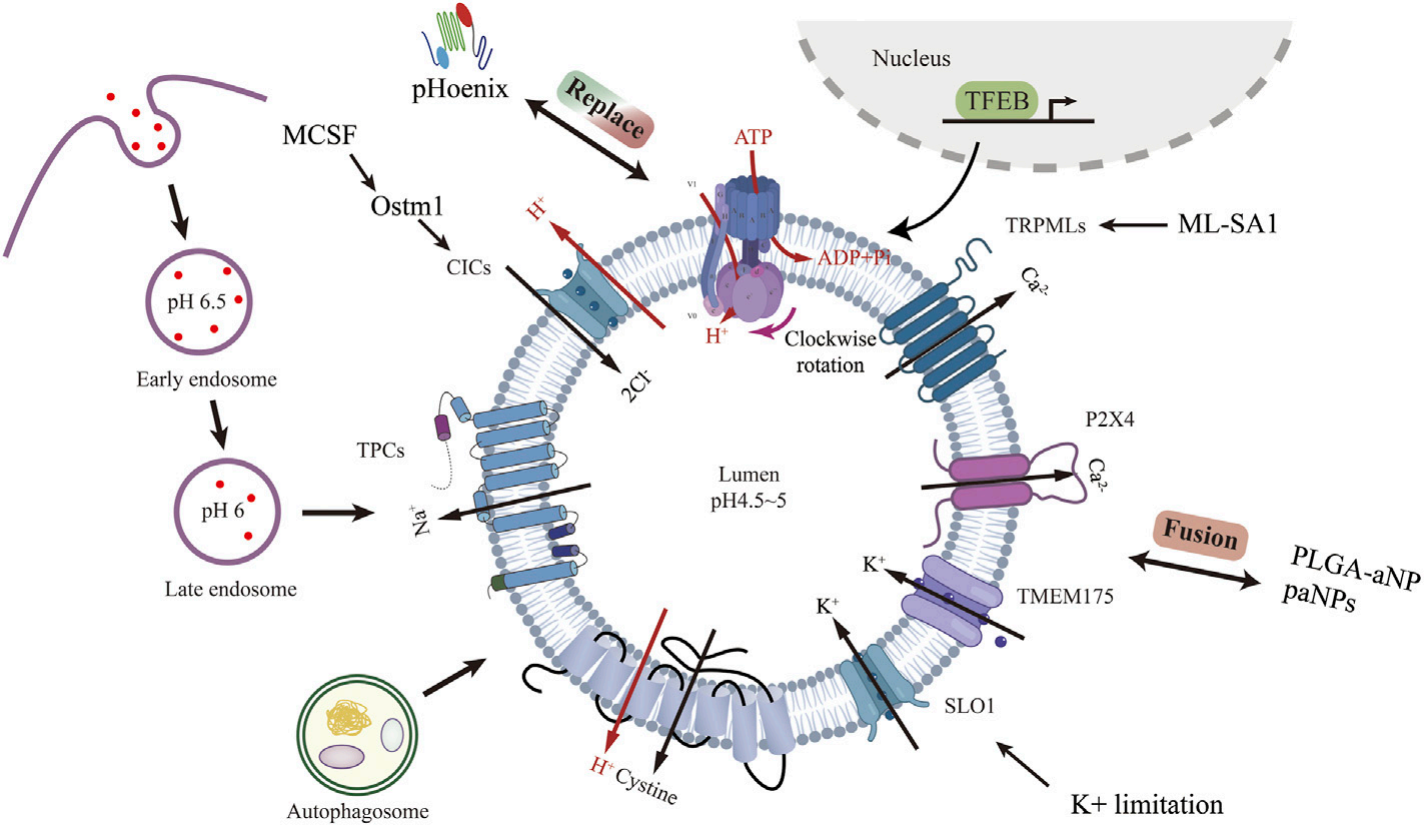
Lysosomal acidification process and regulatory strategies targeting channel proteins other than V-ATPase. Created with BioRender.com.

Vacuolar H^+^-ATPases are ATP-driven pH-regulated proton pumps. V-ATPase was first found in the vesicles of microsomal membrane fractions of maize coleoptiles in 1980 by A Hager et al. (Hager et al., 1980). S Ohkuma et al. first identified v-ATPase on mammalian cell lysosomes in 1982 (Ohkuma et al., 1982). V-ATPase consists of two functional domains, V0 embedded in the lysosome membrane, responsible for proton translocation, and V1 in the cytoplasm, responsible for ATP hydrolysis. Membrane-bound V0 consists of six subunits (a, c, c’, c’’, d, e) and intracellular V1 consists of eight subunits (A, B, C, D, E, F, G, H), several of these subunits are present in multiple copies. Subunit a of V0 accepts and expels protons with the help of a central proteolipid ring consisting of the c, c’, and c’’ subunits. Subunits A, B, and D of V1 form a catalytic core that is involved in the binding and hydrolysis of ATP, while the other subunits play structural and regulatory roles. ATP-driven V1V0 proton transport maintains organelle, cellular and extracellular pH homeostasis (Figure 1). Recently, overexpression of v-ATPase components has been reported to increase lifespan (Hughes and Gottschling, 2012; Ruckenstuhl et al., 2014). However, these were found experimentally in yeast, and whether this is the case in mammals needs further study.

Lysosomal ion channels and transport proteins play a crucial role in lysosomal homeostasis. Lysosomal function requires the maintenance of intraluminal ion homeostasis and membrane potential ΔΨ (defined as V_cytosol_—V_lumen_) (Bertl et al., 1992). But it is not well known how ionic conductance determines ΔΨ. Studies have revealed several lysosomal channels/transporters Cl−, H+, Ca2+, and Na+,K+. Among the counterion channels, CIC and TRPML are the most thoroughly studied. Shigekuni Hosogi et al. found that lowering the level of Cl-leads to lysosomal acidification disruption and dysfunctional autophagy (Hosogi et al., 2014). Mihyun Bae et al. stimulated TRPM1 with agonists, resulting in increased calcium efflux, luminal acidification, and a clear increase in sphingomyelin and Aβ in lysosomes (Bae et al., 2014). Chunlei Cang et al. identified the K+ channel protein TMEM175 on the lysosome, and the stabilization of K+ helps maintain the pH stability of the lysosome during cell starvation (Cang et al., 2015).

Lysosomal biogenesis is mainly regulated by TFEB (Sardiello et al., 2009; Settembre et al., 2011). Translocation of TFEB from the cytoplasm to the nucleus upregulates v-ATPase and lysosomal gene expression. Because of its physiological importance, V-ATPase is seen as the product of housekeeping genes expressed continuously (Wechser and Bowman, 1995). V-ATPase activity can be regulated in a variety of ways. V-ATPase transcription can be enhanced through TFEB-dependent (Peña-Llopis et al., 2011) and non-TFEB-dependent pathways (Zhu et al., 2017). V-ATPase reversible catabolism and recombination have been reported to be regulated by many factors. The formation of disulfide bonds between cysteine residues at the catalytic site of the V-ATPase is another mechanism proposed for regulating the activity of the V-ATPase *in vivo* (Forgac, 1999). Finally, because V-ATPases are electrogenic, parallel ion conductance must accompany proton transport for significant acidification to occur. The regulation of these ion channels to achieve lysosome acidification represents a very comprehensive method that has not been studied. Lysosomal acidification is essential to maintain normal cellular function. Defective lysosomal acidification is a pathophysiological mechanism in a variety of diseases including virus infection (Jouve et al., 2007; Singh et al., 2021), autoimmune disorders (Monteith et al., 2016), neurodegeneration diseases (Lee et al., 2010) and tumor drug resistance (Chauhan et al., 2003). Pathogens avoid phagocytosis by preventing vacuolar acidification (Pujol et al., 2009). Recently, lysosome pH elevation has been found in and MSCs (Wang et al., 2014; Wang et al., 2018a), Lihong Wang and Fang-Wu Wang et al. use acridine orange and Lysosensor™ Green DND-189 to identify the lysosome acidity and activity. Under a confocal laser scanning microscope, they both down-regulated in senescent MSCs.

## Role of Lysosomal Acidification Dysfunction in Cellular Senescence

The proliferative potential of bone marrow MSCs cultured *in vitro* is very limited (Stenderup, 2003), and the presence of aging stem cells severely limits their clinical therapeutic effects. The causes of aging are very complicated, and many studies are still at the hypothetical stage. They include genetic determination theory (Larsson, 2011), oxidative free radical damage theory (Harman, 1956), telomere clock theory (Olovnikov, 1996), metabolic waste accumulation theory (Benveniste et al., 2019), inflamm-aging theory (Franceschi et al., 2000), and so on. In recent years, the relationship between cellular senescence and lysosomal function has received increasing attention (Ansari et al., 2021). Hui Sun et al. found that many lysosomal genes showed differences in aging MSCs (Sun et al., 2021). During aging, lysosomes undergo various modifications that weaken their degradability and increase their susceptibility to metabolic conditions. Impaired lysosomal acidification during cellular senescence is a phenomenon that has been studied and confirmed (Colacurcio and Nixon, 2016). However, whether in turn lysosomal acidification impairment is a determinant of aging remains an open question. Lysosomal acidification disorders have many negative effects on cells, and these effects are highly consistent with the oxidative free radical damage theory of aging and the metabolic waste accumulation theory.

### Defective Cellular Clearance and Accumulation of Toxic Proteins

**FIGURE 2:**
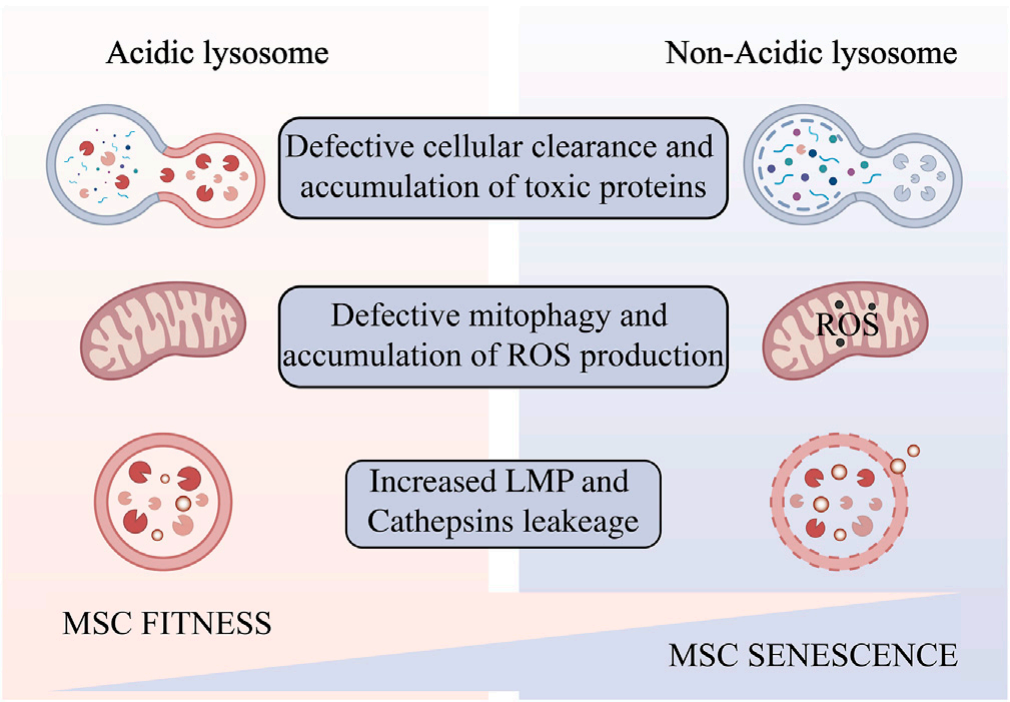
Impaired lysosomal acidification occurs in aging MSCs amplified *in vitro*. Lysosomal alkalinization leads to reduced autophagic flux and accumulation of toxic products, resulting in oxidative stress and increased lysosomal permeability, and ultimately cell senescence and death. Created with BioRender.com.

An abnormal increase in lysosomal pH can have a broad impact on lysosomal digestion. Lysosome alkalization inhibits acidic hydrolases and increases the activity of neutral hydrolases. This shift promotes poor digestion and atypical cleavage of the substrate, which may produce toxic digestion products. Impaired substrate clearance is one of the key lysosomal functions that may be affected by acidification defects (Colacurcio and Nixon, 2016). Altered lysosomal pH may also promote lipid oxidation and ROS generation (Yokomakura et al., 2012). Dan L. Li et al. use dihydroethidium (DHE) staining revealed that knocking down the V-ATPase subunit ATP6V0D1 or ATP6V1B2 in neonatal rat ventricular myocytes (NRVMs) increased cellular reactive oxygen species (Li et al., 2016). This further weaken the integrity of lysosomal membranes (Kurz et al., 2008a), increase the release of cathepsins, leading ultimately a “lysosomal pathway of apoptosis” (Guicciardi et al., 2004) or “lysosomal cell death” (Gómez-Sintes et al., 2016) (Figure 2). The degree of oxidative stress determines the degree of lysosomal membrane instability (Kurz et al., 2008b). In addition, ROS may promote lysosome membrane permeabilization (LMP) by activating lysosomal Ca2+ channels (Sumoza-Toledo and Penner, 2011) or by altering the activity of lysosomal enzymes such as phospholipase A2 (PLA2). Mild LMP activates apoptotic pathways, while extensive LMP can lead to uncontrollable necrosis (Kågedal et al., 2001). Decreased cellular component turnover and accumulation of abnormal intracellular macromolecules are common features of all aging cells. Lysosome-mediated activation of selective-autophagy actively inhibits cellular senescence through degradation of the senescence regulator GATA4 (Kang and Elledge, 2016). Autophagy includes nucleation, autophagosome formation, and fusion with lysosomes (Ktistakis and Tooze, 2016). Each step can be regulated to enhance the autophagy flux and new evidence suggests that autophagic activity can be enhanced by enhancing lysosomal acidification to delay cellular degeneration (Zhu et al., 2017).

### Decreased Mitophagy and ROS Accumulation

During aging, changes in mitochondrial structure and function are evident in most eukaryotes (Seo et al., 2010), but how this occurs is unclear. Adam L. Hughes et al. identified a functional link between lysosome-like vacuoles and mitochondria in *Saccharomyces cerevisiae*, and showed that mitochondrial dysfunction in replicative senescent yeast is caused by altered vesicle PH. Preventing the vacuolar acidity decrease inhibits mitochondrial dysfunction and extends lifespan (Hughes and Gottschling, 2012). In addition, Mikako Yagi et al. found that HIF1α-Nmnat3-mediated NAD (+) levels affected by mitochondrial dysfunction are essential for lysosomal maintenance (Yagi et al., 2021). Mitochondrial ROS production damages lysosomes (Demers-Lamarche et al., 2016). King Faisal Yambire et al. showed that inhibition of lysosomal acidification triggers cellular iron deficiency, which leads to impaired mitochondrial function and cell death (Yambire, 2019). Interplay between lysosome and mitochondrial play an important role in cellular senescence in multiple ways.

## Preventing Lysosomal Acidification Dysfunction and Senescence in MSCs

Pretreatment of MSCs with hypoxia (Lan et al., 2015; Martinez et al., 2017; Sivanathan et al., 2017), oxidative stress (Sharma et al., 2008; Pendergrass et al., 2013), heat shock (Wang et al., 2009; Bolhassani et al., 2019), starvation (Moya et al., 2017), or inflammatory biological agents (Klinker et al., 2017; Boland et al., 2018) has been reported to potentially improve their survival and potency. However, less attention has been paid so far to investigate the potential of directly targeting the lysosomes of MSCs with small molecules, peptide drugs, and nanomaterials. Vacuolar H^+^-ATPase (v-ATPase) defects are the underlying cause of several human diseases (Haggie and Verkman, 2009; Halcrow et al., 2021). New studies have shown v-ATPase activity is altered, and lysosomal pH regulation is dysregulated during cellular senescence and apoptosis (Nilsson et al., 2006). Regulation of lysosomal acidification is an emerging direction in MSCs-based therapy.

### Coupling Efficiency of V-ATPase Pump

Coupling efficiency of the V-ATPase pump is thought to regulate intracellular acidification (Kane, 2006). Interestingly, the assembly of the V0 and V1 structural domains is dependent on the nutrient (Cotter et al., 2015). Amino acid starvation has been shown to promote v-ATPase assembly (Onishi et al., 2019) by inactivating mTORC1 in a TFEB-dependent manner (Peña-Llopis et al., 2011). AKT1, AKT3 isoforms are required to maintain V-ATPase activity in a state of amino acid starvation (Collins et al., 2020). Ju-Hyun Lee et al. showed that presenilin-1 (PS1) knockout impairs the orientation of v-ATPase V0a1 subunit to the lysosome (Lee et al., 2010). Michael C. Jaskolka et al. found that the prokaryotic RAVE and eukaryotic Rabconnectin-3 complexes facilitate the recombination of V1 with V0 during glucose recovery and consequently restore ATP-driven proton transport (Jaskolka et al., 2021). Qing Tang et al. reported the absence of the N-deacetylase and N-sulfotransferase 3 (NDST3) promotes the assembly of the V-ATPase holoenzyme on the lysosomal membrane (Tang et al., 2021). Limor Avrahami et al. found that treatment with a novel substrate competitive GSK-3 inhibitor, L803-mts, restored N-glycosylation of the V-ATPase V0a1 subunit and the impairment caused by dysfunctional presenilin-1 in Alzheimer disease patients (Avrahami and Eldar-Finkelman, 2013; Avrahami et al., 2013). Ju-Hyun Lee et al. reported that isoproterenol (ISO) and related β2-adrenergic agonists re-acidify lysosomes in PSEN1 knockout (KO) cells by restore delivery of vATPaseV0a1 to lysosome (Lee et al., 2020). Youn-Sang Jung et al. found that transmembrane protein 9 (TMEM9), a regulator of vesicle acidification, binds to V-ATPase and promotes its assembly, leading to enhanced vacuolar acidification and trafficking (Jung et al., 2018).

**FIGURE 3:**
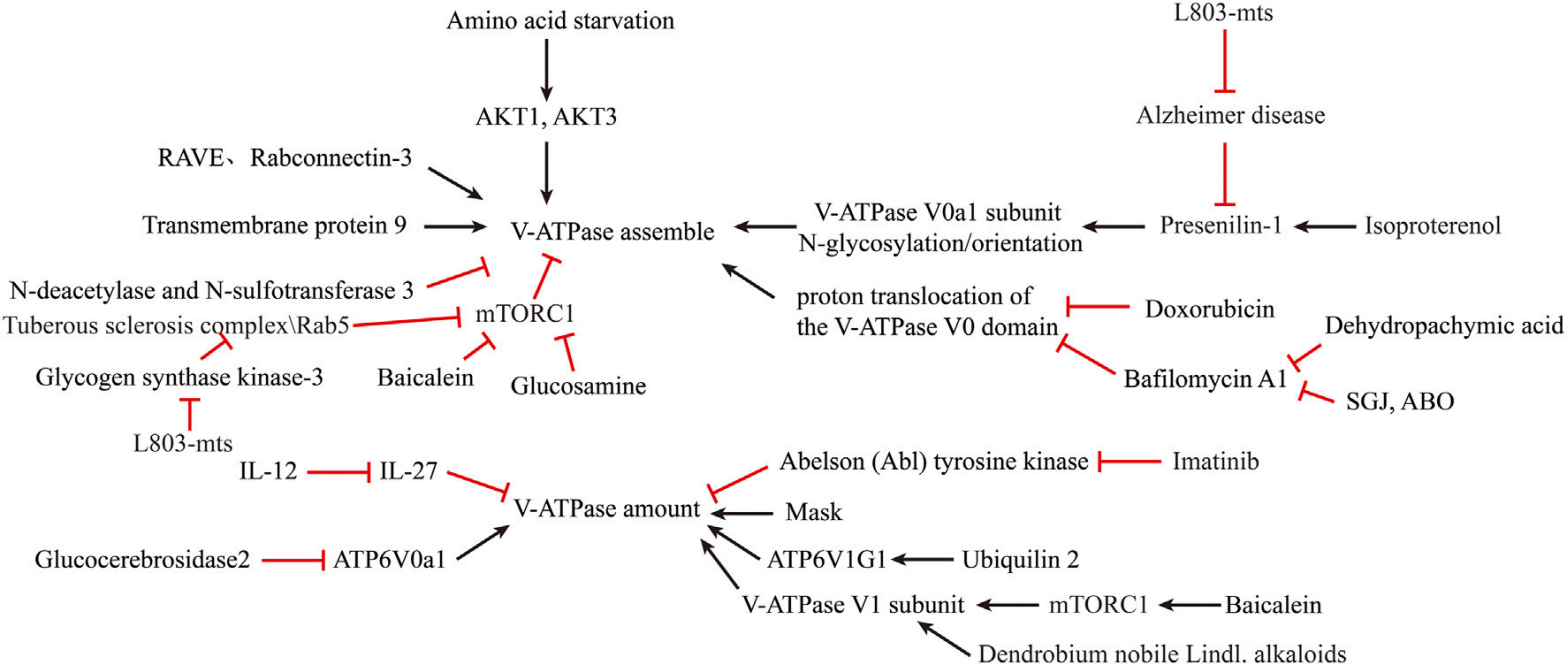
Reversal of lysosomal acidification in *in-vitro* amplified senescent MSCs by regulating V-ATPase activity.

Dan L Li et al. found that Doxorubicin obstructs proton translocation of the V-ATPase V0 domain and impaires lysosomal acidification in cardiomyocytes (Li et al., 2016). Yoshinori Tanaka et al. found that Progranulin (PGRN), a secreted lysosomal protein, promoted lysosomal acidification by enhancing the function of V-ATPase rather than its amount (Tanaka et al., 2017). Limor Avrahami et al. found that inhibition of glycogen synthase kinase-3 (GSK-3) and activation of tuberous sclerosis complex (TSC) promotes lysosomal acidification through the mTORC1/autophagy axis and endocytic trafficking pathways (Avrahami et al., 2020). In contrast, some clinical agents have been found to inhibit lysosomal acidification. Yong Lin et al. shown that GlcN, a dietary supplement widely used to promote joint health and effectively treat osteoarthritis, inhibits the acidification of lysosomes through its amino group (Lin et al., 2020). Modulation of V-ATPase activity is by far the most used method to alter lysosomal acidity (Figure 3).

### Expression and Amount of V-ATPase Pump

A second method to increase lysosomal acidification is to modulate V-ATPase expression. Simon Wheeler et al. reported that inhibition of non-lysosomal glucocerebrosidase2 (GBA2) increased expression of ATP6V0a1 subunit in Niemann-Pick type C disease (NPCD) fibroblasts (Wheeler et al., 2019). Joo-Yong Jung et al. reported that providing IL-12 and neutralizing IL-27 increases lysosomal acidification by increasing V-ATPase expression (Jung and Robinson, 2014). The use of imatinib and siRNA to inhibit the expression of Abelson (Abl) tyrosine kinase increased the transcription and expression of v-ATPase and decreased the pH of lysosomes in human macrophages (Bruns et al., 2012). Traditional Chinese medicine is an emerging source for exploring new treatments for lysosomal acidification disorders. Xinhong Zhu et al. showed that supplementation with Baicalein increased the expression of V-ATPase V1 subunit and co-localization of V1 subunit in mouse lysosomes via the mTOR pathway (Zhu et al., 2020). Jing Nie et al. found that Dendrobium nobile Lindl. alkaloids (DN LA) could increase the expression of the A1 subunit of v-ATPase to promote lysosomal acidification (Nie et al., 2018). Mengyao Yu et al. found that Dehydropachymic acid (DPA) treatment restored the bafilomycin A1-induced increase in lysosomal pH (Yu et al., 2017). Mingwei Zhu et al. found that Mask, an Ankyrin-repeat and KH-domain containing protein, enhance lysosomal acidification by promoting V-ATPase expression levels in a TFEB-independent manner (Zhu et al., 2017). Josephine J Wu et al. identified UBQLN2 as an important regulator of ATP6V1G1 expression and stability, and overexpression of UBQLN2 increased acidification of autophagosomes (Wu et al., 2020).

### Nanomaterials and Artificial Small Molecule Compounds

Nanomaterials and artificial small molecule compounds also have great potential in promoting lysosomal acidification. Most of the nanomaterials taken up by cells are concentrated on lysosomes, making the lysosomal compartment the most common intracellular site for nanoparticle sequestration and degradation (Stern et al., 2012). Lihong Wang et al. invented a novel small molecule, 3-butyl-1-chloro imidazo (Caplan, 1991, Pittenger et al., 1999 pyridine-7-carboxylic acid (SGJ), which can promote lysosomal acidification and inhibit hBMSCs senescence (Wang et al., 2018b). Fang-Wu Wang et al. identified a small molecule compound 6-amino-3,4-dihydro-2H-3-hydroxymethyl-1,4-benzoxazine (ABO), which could promote the expression of Annexin A7 (ANXA7) to counteract the damage of lysosomes by Baf-A1, and inhibit the senescence of MSCs (Wang et al., 2014). Mathieu Bourdenx et al. showed that added poly (DL-lactide-co-glycolide) (PLGA) acidic nanoparticles (aNP) (PLGA-aNP) were transported to the lysosomes in human dopaminergic neuroblastoma BE-M17 cells within 24 h, lowering the lysosomal pH. After PLGA-aNP treatment, defective lysosomes are re-acidified and lysosomal function is restored (Bourdenx et al., 2016). Kyle M Trudeau et al. described a photoactivatable acidified nanoparticle (paNPs) that were taken up by lysosomes in INS1 and mouse β-cells and lysosomal acidity and function was enhanced (Trudeau et al., 2016). Jialiu Zeng et al. reported that biodegradable poly (lactic acid-glycolic acid) (PLGA) nanoparticles (NPs) can be localized to the lysosome to reduce luminal pH and restore autophagic flux in insulin-secreting (INS1) β-cells (Zeng et al., 2019). Benjamin R Ros et al. have proposed a new tool, pHoenix, could functionally replace the endogenous proton pump with the light-driven proton pump Arch3, enabling optogenetic control of lysosomal acidification and neurotransmitter accumulation (Rost et al., 2015).

### Other Ion Channels in Lysosome Membrane

In addition to V-ATPase, targeting other channel proteins can also regulate lysosomal acidification. Amitabha Majumdar et al. found that resting microglia expressed only low levels of osteopetrosis-associated transmembrane protein 1 (Ostm1), which impaired lysosomal transport of voltage-gated chloride channel-7 (ClC-7) protein (Lange et al., 2006). Activation of microglia with MCSF increased lysosomal ClC-7 and Ostm1 transcription, leading to increased lysosomal acidification (Majumdar et al., 2011). Anke Di et al. found that the cystic fibrosis transmembrane conductance regulator Cl–channel (CFTR) contributes to lysosomal acidification (Di et al., 2006). Zhiqiang Xia et al. found that ML-SA1, a TRPML agonist, inhibits dengue virus (DENV) and Zika virus (ZIKV) *in vitro* by promoting lysosomal acidification (Xia et al., 2020). Liang Hui et al. found that TRPML1 agonist ML-SA1 blocked LDL-induced increases in intra-neuronal and secretory levels of Aβ and the accumulation of Aβ in endolysosomes and increase lysosomal acidification (Hui et al., 2019). Huikyong Lee et al. reported that ZnT3/H+/K + -ATPase is another pathway for lysosomal acidification, cAMP activation of PKA increased the overall level and proportion of H+/K + - atpase in lysosomes when v-ATPase is blocked, indicate a potential strategy to overcome this lysosomal dysfunction (Lee and Koh, 2021). Arjun N Sasikumar et al. used a yeast model to show that potassium limitation enhances lysosomal acidity and brings health benefits early in life (Sasikumar et al., 2019).

## Discussion

In this review, we summarize the effects of lysosomal acidification disorders that can further cause cellular senescence and summarize existing strategies for controlling lysosomal acidification disorders. During aging, lysosomal acidification becomes impaired and the luminal pH increases. To begin, a rise in luminal pH decreases acidic hydrolase activity while increasing neutral hydrolase activity. This shift causes inefficient substrate degradation and a rise in hazardous metabolites, as well as a decrease in mitophagy and an increase in ROS generation. ROS accumulation further causes LMP and cathepsins leakage, ultimately leads to cellular senescence and apoptosis. In the next place, we summarize current studies that promote lysosomal acidification in the hope of providing some insights into reversing aging of MSCs. Genetic engineering, traditional Chinese medicine, nanomaterials, and small molecule compounds all have potential to be strategies for lysosomal acidification therapy.

However, the current understanding of lysosomes may still be only the tip of the iceberg. Lysosomal membranes contain hundreds of integrins and peripheral proteins, many of which have unknown functions (Ballabio and Bonifacino, 2020). The upregulation of V-ATPase activity and lysosomal acidification can be beneficial in some diseases as well as harmful in others. Activating only the V-ATPase activity in specific cells without affecting others is also a major difficulty in the clinical transformation process. For all these reasons, the study of lysosomes remains a highly specialized field. More research is needed in the future to focus on the translation of lysosomal biology to clinical applications.
